# The role of growth hormone in assisted reproductive technology for patients with diminished ovarian reserve: from signaling pathways to clinical applications

**DOI:** 10.3389/fendo.2025.1551126

**Published:** 2025-04-17

**Authors:** Peina Han, Huishu Xu, Yuying Yuan, Zheling Wen, Jing Yang, Lei Han, Dongmei Zhang

**Affiliations:** ^1^ Department of Reproductive Medicine, Binzhou Medical University Hospital, Binzhou, China; ^2^ Department of Obstetrics Medicine, Linquan County People’s Hospital, Fuyang, Anhui, China; ^3^ Obstetrics and Gynecology of Binzhou Medical University, Binzhou, Shandong, China

**Keywords:** diminished ovarian reserve, growth hormone, oxidative stress, signaling pathway, reactive oxygen species

## Abstract

Diminished Ovarian Reserve (DOR) is a complex etiology disease that significantly impacts female fertility, endocrine function, and overall health status. In recent years, the incidence of DOR has been increasing, yet therapeutic methods remain relatively limited, particularly for patients with reproductive needs who often require Assisted Reproductive Technology (ART) treatments. Growth Hormone (GH), a peptide hormone secreted by the anterior pituitary, promotes growth in bones, viscera, and multiple organs and systems throughout the body, enhances protein synthesis, and influences fat and mineral metabolism, playing a crucial role in human growth and development. Its levels decrease with the aging of the organism. In recent years, studies have suggested that a decline in growth hormone levels may be one of the causes of decreased ovarian function, leading to the application of GH in assisted reproductive treatments for patients with DOR. An increasing body of research indicates that GH can improve ovarian function through mechanisms such as antioxidant stress, promotion of follicle development, and enhancement of oocyte quality, and it also shows potential to improve endometrial receptivity, making GH a promising safe and effective strategy in ART for DOR patients.

## 1 Introduction

DOR is characterized by a reduction in the number and/or quality of oocytes, leading to insufficient ovarian function, sex hormone deficiency, and decreased fertility. Auxiliary examinations reveal decreased levels of anti-Mullerian hormone (AMH), reduced antral follicle count (AFC) (AMH < 1.1 ng/ml or AFC < 7), and elevated follicular stimulating hormone (FSH) levels (1). A subset of DOR patients suffer from premature ovarian insufficiency (POI) (2). Studies indicate that the prevalence of DOR in the population is approximately 0.9% to 3%, with incidence rates before the ages of 20, 30, and 40 being 0.01%, 0.1%, and 1%, respectively, showing a trend of increasing prevalence and a shift towards younger ages (3). Among women undergoing assisted reproductive technology treatments, the prevalence of DOR is 19% to 26% (4). The etiology of DOR is complex and not fully understood, but it may be related to aging, autoimmune conditions, genetic factors, iatrogenic damage, lifestyle habits, environmental factors, and psychological influences (1). Compared to other organs, the ovaries show more pronounced age-related changes, with a decrease in follicle number, accelerated follicle atresia, and reduced follicle quality, accompanied by a lower probability of natural pregnancy, making it a significant cause of female infertility (5). Therefore, delaying or intervening in the onset of DOR is crucial for improving female fertility rate. In recent years, clinical research on the application of GH has gradually expanded from pediatric growth and development to adult diseases. Studies have shown that GH can delay ovarian aging and effectively treat DOR patients, increasing the success rate of assisted reproductive technologies (6). This article provides a comprehensive review of the application and research advancements of GH in DOR, POI, and its terminal stage, Premature Ovarian Failure (POF).

## 2 An overview of GH and its receptor

**Figure 1:**
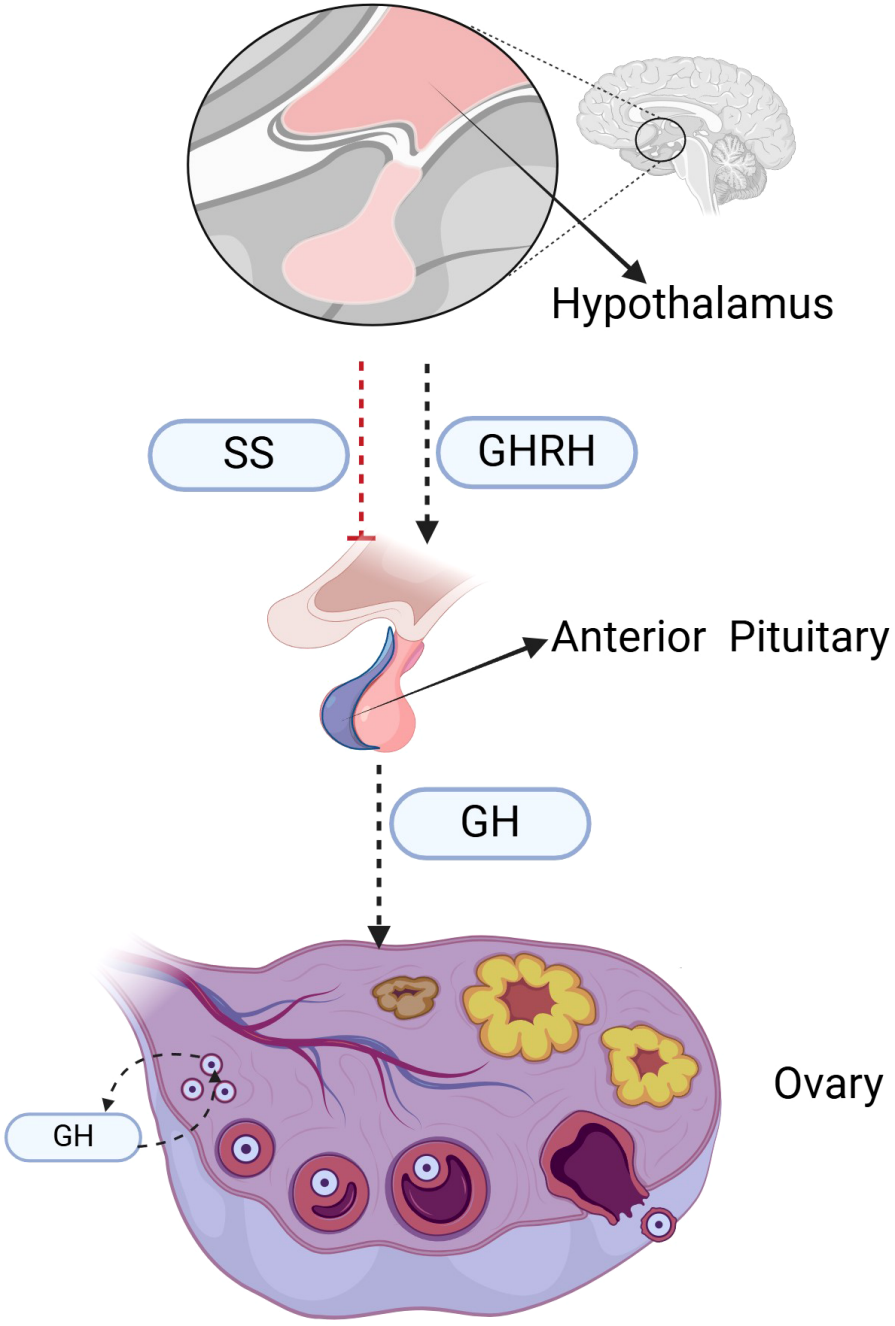
The role of GH in ovarian follicular development within the HPG Axis. The figure illustrates the macroscopic processes in the HPG axis, including the promotion of GH secretion by GHRH, the inhibition of GH secretion by SS, and the role of GH in follicle development. SS, somatostatin; GHRH, growth hormone releasing hormone; GH, growth hormone.

GH is a polypeptide synthesized and secreted by the acidophilic cells of the anterior pituitary gland, predominantly secreted during nocturnal sleep. Comprising 191 amino acids (7), GH is regulated by positive and negative feedback mechanisms involving growth hormone-releasing hormone (GHRH) and somatostatin (SS), respectively. GHRH, produced by the hypothalamus, stimulates the synthesis and release of GH by the acidophilic cells of the anterior pituitary, while SS, also hypothalamic in origin, inhibits GH secretion through receptor binding on these cells, as shown in **Figure 1**. In most instances, GH released by the anterior pituitary into the bloodstream promotes the synthesis and secretion of insulin-like growth factor-1 (IGF-1) in the liver, thereby exerting its physiological functions. The primary functions of GH include: (1) Promotion of growth and development: by stimulating the proliferation and differentiation of osteoblasts and chondrocytes, thereby increasing the growth velocity of long bones; (2) Regulation of the body’s material metabolism: facilitating the breakdown of triglycerides in adipose tissue; modulating the activity of lipoprotein lipase and the number of LDL receptors; enhancing protein synthesis and metabolism; increasing the absorption of skeletal minerals, thereby improving bone density and quality; (3) Modulation of physiological functions: augmenting mitochondrial ATP synthesis in muscle cells; adjusting myocardial contractility and skeletal muscle strength; promoting neural synaptic growth and cognitive function improvement; and elevating nitric oxide levels to increase sexual desire (8).

**Figure 2:**
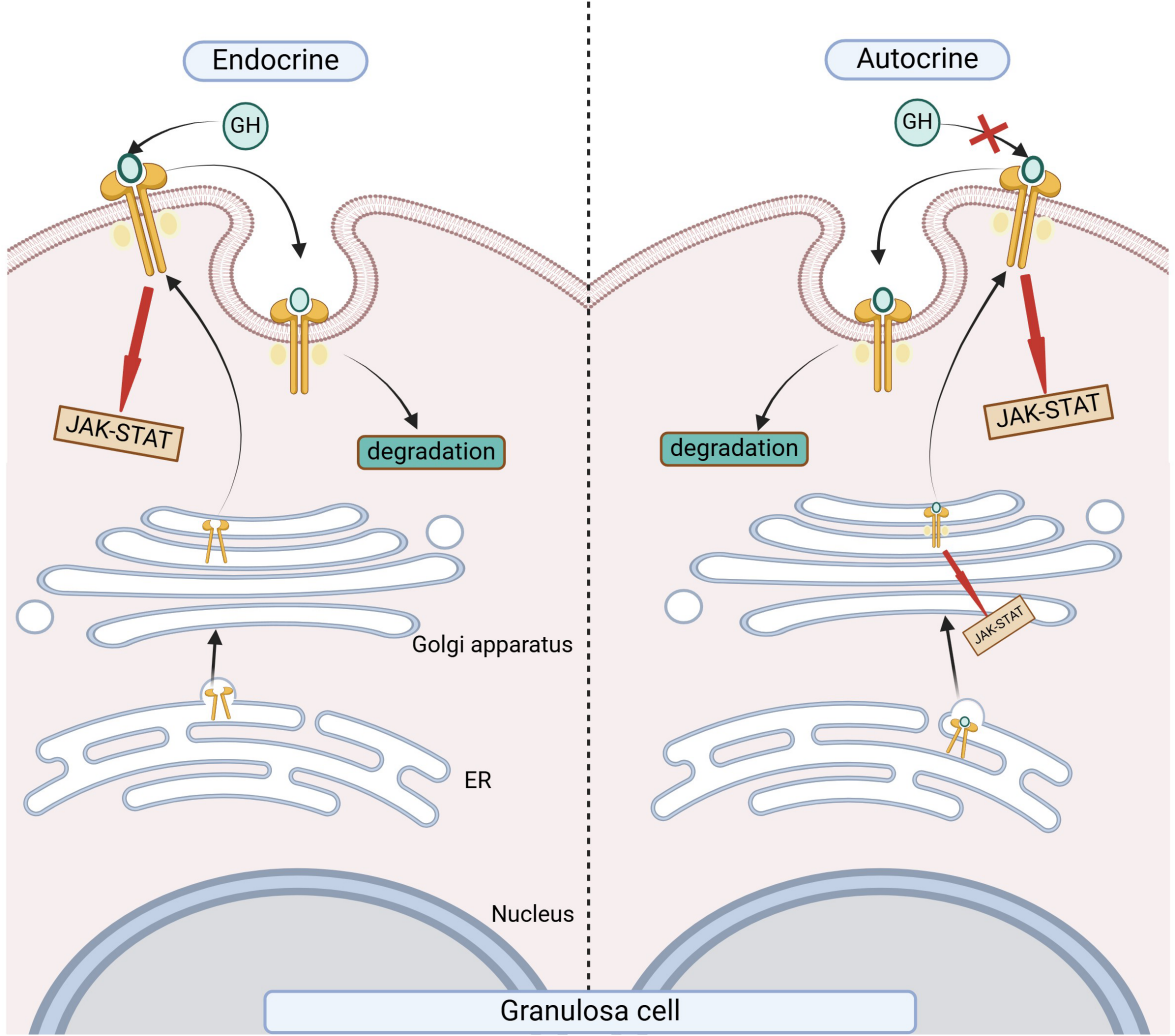
The Process of ovarian autocrine GH secretion. The figure illustrates the comparison between the autocrine and paracrine processes of growth hormone. ER, endoplasmic reticulum.

GH molecules can directly interact with two growth hormone receptor (GHR) molecules on the surface of target cells, simultaneously recognizing and binding to form a GH-GHR2 ternary complex, which activates various intracellular signaling proteins (JAK-STAT, MAPK, etc.), promoting mitosis and growth development (9). However, research has found that the primary biological role of pituitary-derived GH in the body is to act on GHRs on the surface of liver cells, stimulating hepatocytes to produce IGFs (IGF-1 and IGF-2) to exert its function. Among them, IGF-1 promotes the growth of bones, viscera, and the whole body, and stimulates cell proliferation; IGF-2 mainly regulates development during the perinatal period. Subsequent studies have shown that not only the pituitary gland can secrete GH, but female reproductive system organs and tissues such as the ovaries can also locally secrete GH (10, 11). Pituitary secretion of GH is pulsatile, while locally produced GH is characterized by continuous low concentration secretion in the locality, with paracrine and autocrine regulatory modes. GHRs have been identified in granulosa cells, theca cells, oocytes, and in testicular tissue, as well as in the glandular cells of the endometrium and decidua during the luteal phase. This distribution suggests that GH plays a significant role in maintaining the function of female reproductive organs and tissues, including the ovaries and uterus (12, 13). **Figure 2** illustrates that GH synthesized by the ovary itself is produced on the endoplasmic reticulum (ER) and rapidly binds to GHR within the ER, promoting the maturation of GHR and forming a GH-GHR complex. These complexes then activate the JAK-STAT signaling pathway through the Golgi apparatus system. Once the complex moves to the cell surface, autocrine GH induces the JAK-STAT signaling pathway; however, signal transduction does not occur when GHRs are still within the ER. This process indicates that activation of the JAK-STAT pathway is not necessarily contingent upon binding to the extracellular domain of the cell membrane GHR (14, 15). The majority of patients with DOR are related to increasing age and disease onset. As age advances, there is a decline in the function of the adenohypophysis and ovaries, which inevitably leads to a decrease in reproductive function alongside a reduction in GH levels. This provides a theoretical basis for the therapeutic use of exogenous GH in the treatment of DOR and in the field of assisted reproduction.

## 3 Mechanisms of DOR and the impact of GH on follicles

### 3.1 Pathophysiological mechanisms underlying the development and progression of DOR

The physiological processes through which the ovaries perform their reproductive functions primarily include follicular growth, maturation, ovulation, and corpus luteum formation. The development of primordial follicles to maturity involves four stages: primordial, primary, secondary, and mature follicles. The size of the primordial follicle pool represents the reserve of fertility potential, with early activation of primordial follicles determining the ovarian reserve function and serving as the foundation for follicular development. The vast majority of primordial follicles remain in a quiescent state, with only a small fraction initiating growth, eventually maturing and ovulating (16). Possible causes of DOR include depletion of the follicle pool, increased follicular atresia, and reduced sensitivity to gonadotropins (Gn), leading to a decline in ovarian function (17). Patients with DOR often exhibit excessive reactive oxygen species (ROS) production in ovarian tissue, leading to an imbalance between oxidation and antioxidation, which results in apoptosis of ovarian granulosa cells, mitochondrial dysfunction, inflammation, and telomere shortening (18). A prospective clinical study by Yan Huang et al. indicates that compared to individuals with normal ovarian reserve (NOR), DOR patients have significantly increased oxidative stress products and inflammatory markers in follicular fluid, along with reduced antioxidant status, suggesting that interventions targeting oxidative stress may potentially improve DOR (19).

### 3.2 Growth hormone-stimulated activation of primordial follicles and follicular development

The Hypothalamic-Pituitary-Ovarian (HPO) axis regulates the secretion of steroid hormones through Gn, thereby influencing follicular development. Patients with DOR exhibit elevated follicle-stimulating hormone (FSH), decreased estradiol (E2) and GH levels, and impaired follicular development, with GH capable of modulating these changes. Although pituitary-derived Gn are the primary regulators of ovarian steroidogenesis, *in vitro* studies have shown that GH can also promote follicular development and maturation, as well as ovulation, by binding to receptors on ovarian granulosa cells (GCs) and thecal cells (TC). Additionally, GH indirectly modulates the production of E2 and progesterone (P) by GCs through the stimulation of IGF-1 production in the liver and ovaries (11, 20). In bovine granulosa cells and luteinized human granulosa cells, growth hormone (GH) can increase the production of estradiol (E2) and progesterone, thereby promoting follicular development (15, 21). Additionally, during the early stages of follicular development, the activation of primordial follicles and their transformation into antral follicles is independent of Gn, with this process being modulated by various locally produced growth factors through paracrine or autocrine mechanisms (22). Saccon et al. employed GH-deficient Ames Dwarf (df/df) mice to block GH secretion at the pituitary level, leading to a significant increase in the number of primordial follicles compared to Normal (N/df) mice and wild-type mice. In contrast, the number of follicles in developmental stages and mature follicles markedly decreased. Reduced GH secretion is correlated with an increase in follicular atresia, a condition that can be reversed by the exogenous supplementation of GH. This indicates that GH may be involved in the activation of primordial follicles and plays a pivotal role in stimulating follicular development to the gonadotropin-dependent phase (23). In GHR gene-knockout mice, circulating GH levels are high while IGF-1 levels are low. Histological examination of the ovaries reveals an increase in the number of primordial or primary follicles and a decrease in the number of healthy and growing antral or preovulatory follicles. This suggests that in the absence of GH, follicles remain in the primordial stage (24). A previous animal experiment involved the use of transgenic mice expressing Bovine Growth Hormone (bGH) under the control of the phosphoenolpyruvate carboxykinase (PEPCK) promoter, which were observed alongside untreated normal mice under identical conditions for 15 days. It was found that the ovulation rate in transgenic mice was significantly increased. To further assess the impact of bGH on the ovulation rate in non-transgenic mice, an experimental group of non-transgenic mice was injected with either 0.75 mg bGH per day or 0.30 mg bGH per day (as a single dose or 0.15 mg twice daily) for three days. Compared to the control group, the ovulation rate increased in non-transgenic mice treated with 0.75 mg bGH per day, indicating that GH transgenic expression or administration of GH enhances the ovulation rate in mice (25). Additionally, GH may be essential for late follicular development, as GHR deficiency leads to complete inhibition of dominant follicle development in cattle (26). Furthermore, GH can promote the repair and generation of ovarian blood vessels by stimulating the production of vascular endothelial growth factor 1 (VEGF-1), and a richer blood supply is also a significant factor in promoting follicular growth (27). In summary, GH is indispensable in the growth and development of follicles.

### 3.3 GH mitigates ovarian oxidative stress, ameliorates mitochondrial dysfunction, and modulates cell apoptosis

Oxidative stress (OS) and apoptosis are closely associated with the development of DOR in busulfan/cyclophosphamide (Bu/Cy)-induced mouse ovary aging models (28). Apoptosis of oocytes and female germline stem cells (FGSCs) directly leads to a reduction in the number of germ cells, decreasing ovarian reserve and reproductive potential in infertility mice treated with Bu/Cy (29). Extensive apoptosis of GCs reduces the number of primordial follicles, impairs the reproductive capacity of FGSCs, and disrupts the metabolic homeostasis of the ovary, thereby damaging ovarian function on pig CGs (30). Studies have indicated that GCs apoptosis is closely related to the depletion of mitochondrial DNA; mitochondrial dysfunction disrupts the normal production of ATP and increases the levels of Reactive Oxygen Species (ROS), leading to pathological changes (31). Further research has found that an increase in ROS can disrupt mitochondrial membrane permeability. Enhanced mitochondrial membrane permeability may be a limiting factor in apoptosis; if it increases, causing mitochondrial matrix swelling, it leads to a decrease in mitochondrial membrane potential and the collapse of the inner membrane potential, a consequent decline in ATP generation, and ultimately, the release of cytochrome C (apoptotic factor) and cell apoptosis. The release of apoptotic factors can further promote an increase in ROS and mitochondrial damage (32). In aged ovaries, an overproduction of ROS leads to cellular structure damage (mitochondria, DNA, proteins, cell membranes, and ER) and cell apoptosis. Therefore, antioxidants may inhibit ovarian OS, improve mitochondrial function and cell apoptosis, thus preventing the occurrence and development of DOR (33). Although GH is not a direct antioxidant, it can intervene in the signaling pathways of cellular defense mechanisms against OS, exerting an antioxidant effect (34).

**Figure 3:**
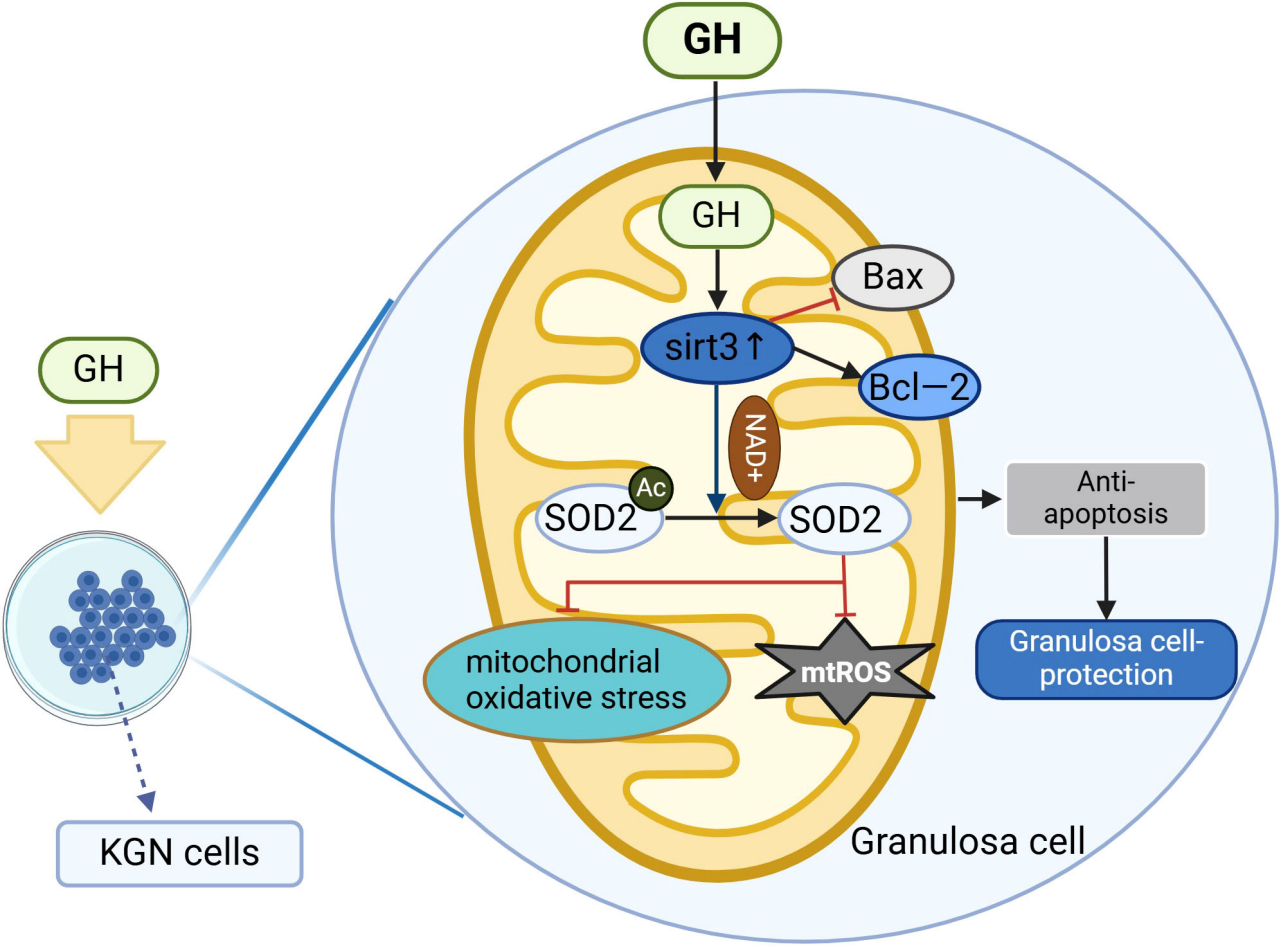
Mechanism of GH protection in GCs. The figure illustrates the regulation of oxidative and antioxidant processes by GH in the mitochondria of KGN cells. Bax, pro-apoptotic protein; Bcl-2, anti-apoptotic protein; mtROS: mitochondrial reactive oxygen species.

Wang et al. treated human ovarian granulosa cell line (KGN cells) with cisplatin to establish an *in vitro* model of GCs apoptosis and mitochondrial dysfunction. Following cisplatin treatment, cell proliferation was significantly inhibited, and the rate of apoptosis increased (P < 0.05). However, treatment with GH rescued cell proliferation (for details, see **Figure 3**), reduced the rate of apoptosis, decreased the ratio of Bax (pro-apoptotic protein) to Bcl-2 (anti-apoptotic protein) (P < 0.05), significantly lowered levels of abnormal ROS, and increased the levels of Sirt3 and Sod2 (antioxidant components), thereby alleviating OS. Additionally, GH was found to increase the mitochondrial membrane potential and the copy number of mitochondrial DNA (mtDNA) in GCs. This suggests that GH can mitigate OS, enhance mitochondrial function, and protect against cisplatin-induced apoptosis in GCs (35).

Studies have shown that cyclophosphamide (CTX) disrupts the microenvironment necessary for oocyte maturation by inducing GCs apoptosis, leading to sustained ovarian dysfunction and suppression of follicle development (36), as well as inducing the activation of pro-apoptotic genes, the production of ROS, and the depletion of glutathione (37). Feng et al. established a mouse model of DOR with CTX and intervened with recombinant human growth hormone (rhGH). The results indicated that high doses of rhGH increased the number of oocytes retrieved, effectively reduced GCs apoptosis, and mitigated ROS and OS induced by superoxides. Furthermore, rhGH regulated the energy metabolism of oocytes by modulating mitochondrial membrane potential and ATP content, but had no effect on the regulation of mitochondrial DNA (mtDNA) copy number. Single-cell transcriptome analysis revealed that rhGH directly or indirectly promoted the balance between oxidation and antioxidation (38). This suggests that the mechanism of GH treatment for DOR may be related to alleviating OS and mitochondrial damage, thereby maintaining and improving the normal development of follicles.

## 4 Signaling pathways associated with GH treatment for DOR

**Figure 4:**
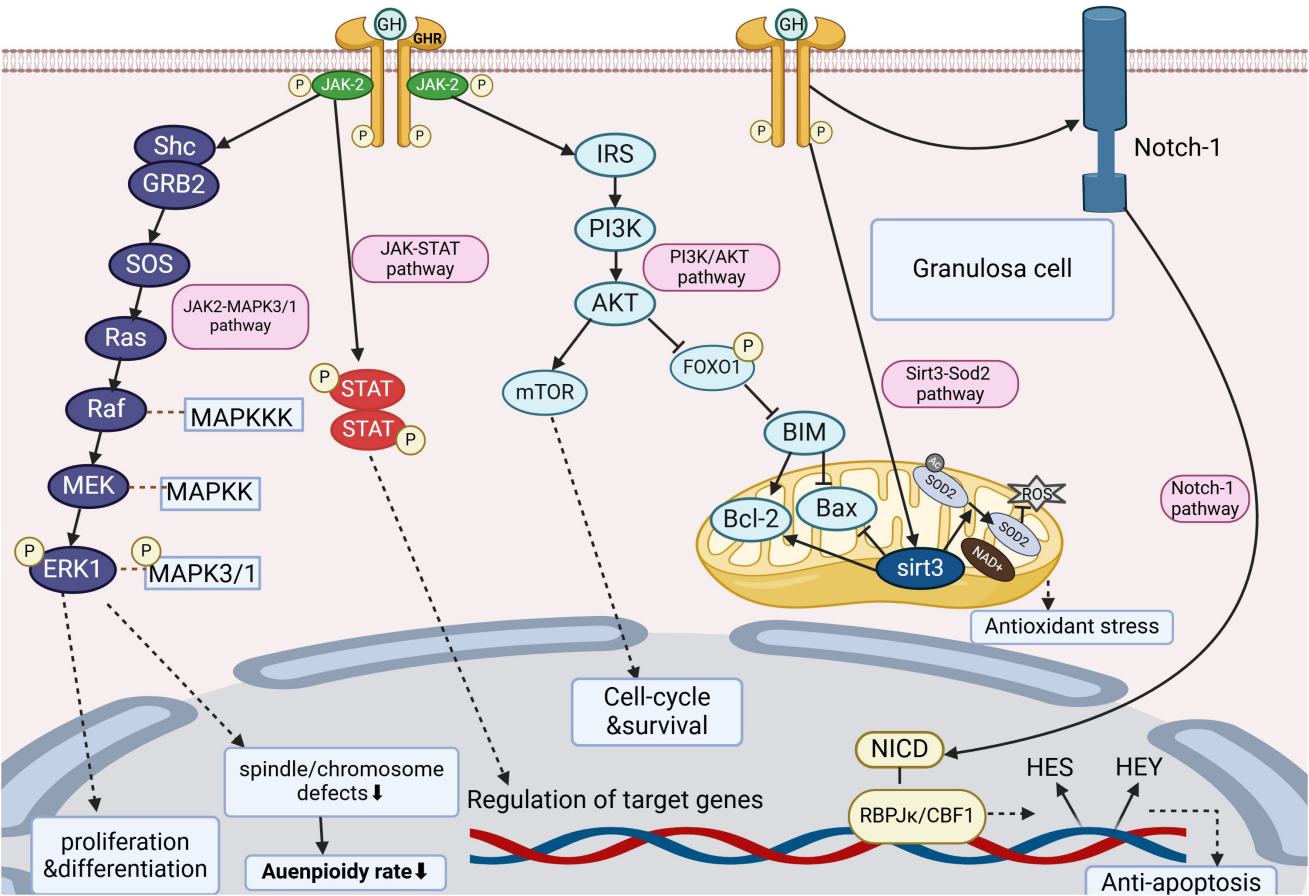
Signaling pathways associated with GH treatment for DOR. The figure illustrates the processes of each signaling pathway in granulosa cells, as well as the corresponding functions they exert.

The mechanism of action of GH in the treatment of DOR involves multiple signaling pathways, which are crucial for follicular development, oocyte maturation, and the maintenance of ovarian function. The following are the main signaling pathways through which GH affects DOR, collectively regulating the complex biological processes within the ovary. The detailed mechanisms and interactions of specific signaling pathways can be visually presented through **Figure 4**.

### 4.1 JAK2-MAPK3/1 signaling pathway

Yonatan B et al. reported that the MAPK signaling pathway is activated during the resumption of meiosis in oocytes, regulating spindle assembly and cell cycle progression, and mediating the physiological effects of Gn in granulosa cells, promoting cumulus expansion, ovulation, and CL formation. It plays a key role in regulating meiotic processes and oocyte aging (39, 40). In mammalian oocytes, the activity of MAPK3/1 is crucial for the progression of the first meiotic division and maintaining the arrest at Metaphase II (MII) (41). Furthermore, increased expression of MAPK3/1 also promotes microtubule organization and spindle assembly during oocyte meiotic maturation (42, 43). Janus kinase 2 (JAK2), a member of the Janus kinase family, plays an important role in signal transduction of growth hormone (44). Additionally, another study pointed out that in mouse oocytes and early cleavage stage embryos, JAK2 is located at chromosome 9p24. Treatment of oocytes with the selective JAK2 inhibitor CEP-33779 was found to significantly reduce the efficiency of oocyte maturation, revealing that JAK2 has a critical role in the maturation process of oocytes (45).

Luo et al. ingeniously combined *in vivo* and *in vitro* studies to investigate the effects of GH on the quality of oocytes in aged mice and its potential link to the MAPK3/1 pathway. They selected 8-month-old aged mice as subjects and administered GH at a dose of 1.6 mg/kg via daily intraperitoneal injection for 8 weeks to simulate a long-term *in vivo* environment of GH supplementation. Concurrently, they collected germinal vesicle (GV) stage oocytes from aged mice and treated them with GH during the *in vitro* simulation of oocyte maturation. These experiments not only revealed that the decrease in p-MAPK3/1 levels in oocytes of aged mice is closely associated with an increase in aneuploidy rates but also suggested that elevating MAPK3/1 activity could be an effective strategy for improving oocyte quality. Furthermore, they found that GH supplementation significantly increased p-MAPK3/1 levels in aged oocytes and correspondingly reduced aneuploidy rates. This finding provides strong evidence for the application of GH in improving oocyte quality. Additionally, GH was able to partially reverse spindle/chromosome defects induced by MAPK3/1 inhibition, thereby enhancing the developmental potential of aged oocytes. The data from this study confirmed that the enhancing effect of GH on p-MAPK3/1 is closely related to the increased expression of p-JAK2 (46). In summary, Luo et al.’s research revealed the important role of the MAPK3/1 pathway in the reduction of aneuploidy in aging oocytes mediated by GH and emphasized the potential value of the JAK2-MAPK3/1 pathway in improving the quality of aged oocytes (46). These findings provide new directions for future research and hold promise for more effective treatment options for DOR patients.

### 4.2 Notch-1 signaling pathway

The Notch signaling pathway is an extremely important and complex network that regulates cell fate, proliferation, differentiation, and apoptosis, thereby playing a key role in tissue development, maintenance of adult homeostasis, and self-renewal of stem cells (47, 48). The Notch proteins are a family of receptors that regulate cell fate. These Notch receptors are activated upon direct contact with ligands on adjacent cells (48, 49). In mammals, there are four Notch receptors (NOTCH1-NOTCH4) and five ligands (Jagged-1 and -2, and Delta-like proteins 1, 3, and 4) (48, 50). NOTCH receptors possess extracellular, transmembrane, and intracellular domains (48, 49). RBPJκ is a key transcription factor in the canonical Notch signaling pathway, acting downstream of all four Notch receptors (49–51). Within the nucleus, the NICD (Notch receptor’s intracellular domain) forms a transcription-activating complex with RBPJκ/CBF1, playing a central role in the Notch signaling pathway (49–51). Activating the transcription of downstream target genes, such as the primarily activated target genes including HES (hairy/enhancer of split) and HEY (Hey-hairy/enhancer-of-split related with YRPW motif family members), which are basic helix-loop-helix (bHLH) family transcription factors. These transcription factors further promote the expression of downstream genes, affecting cell proliferation and inhibiting cell differentiation (51, 52). Additionally, it has been reported that the overexpression of Notch-1 inhibits apoptosis in various types of human cancers, indicating its potential as a therapeutic target for inhibiting cell apoptosis (49–52).

Liu et al. established a POF mouse model using CTX and observed changes in ovarian weight, hormone secretion, and the number of normal follicles in mice treated with recombinant murine growth hormone (rmGH). After treatment with rmGH, there was a significant improvement in the pathological condition of ovarian tissue, a reduction in damage to ovarian granulosa cells, a decrease in the number of atretic follicles, and a significant increase in the number of mature oocytes. Enzyme-linked immunosorbent assays indicated that the plasma E2 levels in rmGH-treated mice increased over time, while plasma FSH levels decreased. The study analyzed the expression levels of genes related to the Notch-1 signaling pathway in mouse ovarian granulosa cells using RT-qPCR and found that medium and high doses of rmGH activated the Notch-1 signaling pathway in the ovarian granulosa cells of POF mice; immunohistochemical staining for Notch1 signaling pathway factors (Notch1, CBF1, and HES1) was positive in the WT, medium dose, and high dose groups, but not in the low dose or saline-treated groups (53). This suggests that GH promotes ovarian tissue repair and regeneration, E2 release, and oocyte maturation by activating the expression of Notch-1 signaling pathway factors in the ovarian tissue of POF mice.

### 4.3 PI3K/AKT signaling pathway

The PI3K-AKT pathway is a critical intracellular signal transduction pathway that responds to extracellular signals and regulates key biological processes such as cell metabolism, proliferation, survival, migration, and angiogenesis. Within this pathway, the PI3K/AKT/mTOR signaling axis is closely associated with the autophagy process in granulosa cells. Specifically, activated phosphoinositol-3 kinase (PI3K) catalyzes the phosphorylation of the metabolite PIP2 into PIP3, which then activates AKT. Recent studies on humans and mice have confirmed that the PI3K/Akt signaling plays an essential role in the regulation of GCs growth and apoptosis during follicular development (54, 55). The forkhead box O (FOXO) family of transcription factors are downstream targets of PI3K/Akt. Phosphorylation of FOXOs by p-Akt inhibits the transcriptional function of FOXOs and contributes to cell survival, growth, and proliferation (56). As a member of the FOXO family, FOXO1 plays a key role in upregulating the expression of downstream pro-apoptotic genes. FOXO1 promotes the activation of the mitochondrial pathway by upregulating Bax and downregulating Bcl-2 expression, thereby mediating GCs apoptosis (57, 58).

GH, by activating the PI3K/Akt signaling pathway, can significantly reduce cell apoptosis induced by OS, an effect that has been confirmed in various cell types, including cardiomyocytes and skeletal muscle cells (59, 60). Therefore, GH shows broad application prospects in the treatment of OS-related pathological processes (60). It is worth mentioning that the GH receptor is not only expressed in various muscle cells but also in human GCs and oocytes, providing a basis for the role of GH in the reproductive system. GH improves oocyte quality and outcomes of *in vitro* fertilization (IVF) by enhancing mitochondrial function (61). An animal experiment by Liu et al. also demonstrated that GH treatment could promote GCs proliferation by reducing OS through the PI3K/Akt signaling pathway. These results indicate that GH treatment can affect ovarian activity conducive to follicle and oocyte development. The aforementioned research findings provide strong theoretical support and practical basis for the targeted treatment of GH in DOR with respect to this signaling pathway (62).

### 4.4 Sirt3-Sod2 signaling pathway

Sirtuins are a class of nicotinamide adenine dinucleotide-dependent deacetylases that participate in numerous key biological processes by reducing the acetylation levels of proteins within cells. Activation of the Sirtuins signaling pathway not only delays the activation of primordial follicles and follicular atresia but also promotes the assembly of the meiotic spindle and chromosome segregation. Additionally, Sirtuins are involved in oocyte DNA damage repair, counteract oxidative stress damage in oocytes, and promote mitochondrial biogenesis. Therefore, Sirtuins are potential targets for the treatment of POF. The Sirtuins family includes multiple members from SIRT1 to SIRT7, each with different subcellular localizations and enzymatic activities, thus playing diverse biological functions in cells (63). Sirtuin 3 (Sirt3), as an important member of the Sirtuins family, is primarily localized in the mitochondria and possesses NAD+-dependent protein deacetylase characteristics. Sirt3 plays an indispensable role in maintaining mitochondrial function and stability. Notably, Sirt3 has a significant impact on maintaining the homeostasis of ROS within the mitochondria (64). In addition to directly reducing ROS production, Sirt3 can also remove the acetylation level of manganese superoxide dismutase (Sod2). Sod2 is activated after deacetylation, further promoting the detoxification of ROS and inhibiting mitochondrial oxidative stress (65).

Wang et al. established an *in vitro* apoptosis model of granulosa cells using cisplatin and observed cell proliferation, apoptosis, ROS, mitochondrial membrane potential, and mtDNA copy number after GH administration. They found that GH could promote the survival and proliferation of granulosa cells and alleviate cisplatin-induced apoptosis in these cells. GH, through the Sirt3-Sod2 pathway, activated Bcl-2 and downregulated Bax, thereby significantly reducing abnormal ROS levels, increasing the degree of mitochondrial membrane potential depolarization, and mtDNA copy number. This study demonstrated that GH protects ovarian granulosa cells from cisplatin-induced apoptosis and enhances mitochondrial function through the Sirt3-Sod2 pathway, reducing OS and apoptosis (35), providing new theoretical basis and therapeutic strategies for the treatment of DOR.

## 5 Impact of GH on oocytes and pregnancy

### 5.1 GH enhances oocyte quality

Oocyte maturation must undergo a series of processes, including nuclear and cytoplasmic maturation, to complete the transition from a gamete to an embryo. This process also involves epigenetics, including post-transcriptional modifications such as gene expression regulation, cell differentiation, and disease occurrence (66). Nuclear maturation encompasses a series of processes from germinal vesicle breakdown, resumption of meiosis, to the release of the first polar body, which reverses the arrest at Prophase I (Pro I) of meiosis and drives meiosis to progress to MII. Cytoplasmic maturation includes the redistribution of organelles, cytoskeletal dynamics, and molecular maturation, which prepares the oocyte for activation and pre-implantation development (67).

GH has beneficial effects on nuclear maturation and oocyte quality. Numerous animal experimental studies have shown that exogenous administration of GH can promote the nuclear maturation process of cumulus-oocyte complexes in various species (horses, dogs, cattle, sheep) (68–71). Previous research has found that GH promotes cell proliferation, inhibits apoptosis, and suppresses the synthesis of connexin 43, thereby promoting cumulus cell proliferation and oocyte maturation. It can also enhance oocyte quality by upregulating oocyte receptors and increasing mitochondrial activity (72, 73). Additionally, GH has shown a promoting effect on cytoplasmic maturation; adding GH to *in vitro* culture systems can significantly increase cortical distribution in equine and bovine oocytes (74, 75). Furthermore, adding IGF-1 to the culture medium of immature oocytes during *in vitro* culture can increase the number of mature oocytes (76) and reduce the number of abnormal oocytes (77). The study by Scheffler et al. demonstrated that the levels of GH and IGF-1 in the follicular fluid of normal oocytes, as well as the fertilization rates, were higher than those in abnormal oocytes, indicating a positive correlation between the GH/IGF-1 content in follicular fluid and oocyte quality (78). This further confirms that the exogenous use of GH and IGF-1 can enhance oocyte quality.

### 5.2 GH improves embryo quality and increases pregnancy rates

A large-scale foreign clinical survey indicates that the incidence of DOR is approximately 19%-26% (4). DOR is one of the significant causes leading to the decline in the success rate of ART. The quality of oocytes and embryos is closely related to clinical pregnancy rates (79). During the early development of embryos, mRNAs, proteins, and mitochondria derived from oocytes are key factors determining implantation (80). Experimental results show that GH has an improving effect on oocyte mitochondrial function, cytoplasmic maturation, and nuclear maturation; therefore, exogenous supplementation of GH can increase the number of mature oocytes obtained, thereby improving embryo quality (81, 82). Previous research found that injecting IGF-1 into the ovaries of cows significantly increased the number of inner cell masses (ICM) during the transition from morula to blastocyst, suggesting that IGF-1 may regulate the development of early embryos (83). Regarding embryo quality, studies on follicular fluid hormones indicate that high levels of GH in follicular fluid are positively correlated with several parameters of embryo implantation potential, including cleavage rate, embryo morphology, embryo development speed, chromosomal abnormalities, zona pellucida factors, and embryo culture environment (84). A randomized controlled trial (RCT) using a long protocol of GnRH agonist showed that GH adjuvant therapy can improve the number and quality of oocytes retrieved, mature oocytes (MII), fertilized oocytes, and the number and quality of embryos for transfer and cryopreservation (85), indicating that GH can not only improve oocyte quality but also embryo quality. A recent meta-analysis including 10 RCTs showed that when the GH dosage was 3IU-5IU, GH was associated with an increase in clinical pregnancy rates (RR=1.63, 95%CI [1.31, 2.03], P<0.0001) (86). An RCT conducted by Li et al. recruited 158 patients who had at least one IVF cycle failure due to a lack of high-quality embryos, using GH 3IU daily, and the results showed that GH supplementation increased implantation rates, pregnancy rates, and live birth rates (87). All the above research results indicate that GH can improve pregnancy rates in DOR patients and increase the success rate of ART.

### 5.3 GH promotion of ovarian angiogenesis and maintenance of corpus luteum function

The ovarian arteries originate from the branches of the abdominal aorta. Although the primary sources of blood supply to the ovaries remain unchanged throughout development, a complex network composed of initially nonfunctional small arteries derived from the ovarian arteries and newly formed capillaries continuously evolves to support the functions of follicular development, ovulation, and the formation of the corpus luteum (CL). Since puberty, the processes of folliculogenesis, ovulation, and the formation and maintenance of CL function are critically dependent on angiogenesis. The ability of the ovaries to maintain normal physiological functions is not only due to Gn but also related to the intervention of GH, which promotes the generation of ovarian blood vessels, thereby further promoting CL development and maintaining CL function (88). In addition, the supportive role of GH on CL function is also demonstrated by its ability to induce the production of P and exert anti-apoptotic effects (89). Studies have shown that during the early stages of bovine CL, the secretion of prostaglandin F2α and P by luteinized granulosa cells is increased through the exogenous addition of GH and other factors, thus maintaining CL function (90).

## 6 Use and dosage of GH in the assisted reproductive process for DOR

Several studies have explored adjuvant therapies that improve ovarian function and increase the success rate of pregnancy. A meta-analysis has indicated that DHEA (dehydroepiandrosterone) and Coenzyme Q10 are two additional adjuvant therapies that improve the likelihood of pregnancy, producing better clinical outcomes in terms of pregnancy success rates and the dosage of Gn required for ovulation induction compared to the control group (91). In 2016, an RCT by Kotb et al., based on the Bologna criteria for defining POR (poor ovarian reserve) patients, showed that DHEA supplementation could improve ovarian responsiveness, reduce the number of days of ovarian stimulation and the total dose of Gn, increase the number of oocytes retrieved, and improve fertilization rates, implantation rates, clinical pregnancy rates, and ongoing pregnancy rates (92). However, an RCT by Kara et al. in 2014 did not find positive results from DHEA supplementation (93). In 2015, a study by Ben-Meir et al. found that in aged mice, ovarian responsiveness was significantly improved during COH (controlled ovarian hyperstimulation) after pretreatment with Coenzyme Q10 (94). However, research on Coenzyme Q10 in human assisted reproduction is still relatively limited.

In 1988, Homburg et al. were the first to utilize GH to promote ovulation, aiming to enhance ovarian response to Gn and thereby increase the number of retrieved oocytes (95). As GH can improve ovarian sensitivity to Gn, increase the number of recruitable follicles, and improve the rate of high-quality embryos, it has been used as an adjunctive therapy during the ovulation induction phase in ART for over three decades (96). With a deeper understanding of the mechanisms of GH action, in recent years, numerous studies have begun to focus more on the application of GH in ART for specific patient populations, particularly those with DOR. He et al.’s study observed that adjunctive GH therapy altered the metabolic profile in follicular fluid of DOR patients, particularly increasing the level of the antioxidant metabolite itaconic acid (97). Zhang et al.’s study, by comparing the impact of different durations of GH pretreatment on the number of retrieved oocytes, found that GH pretreatment could improve treatment outcomes in DOR patients (98). Studies by Chen et al. and Dogan et al. both indicated that GH pretreatment could significantly increase the number of retrieved oocytes and improve embryo quality (99, 100). The study by Haydaredeoğlu et al., unlike most clinical studies using GH alone, performed DHEA and testosterone combined with GH pretreatment, and the results showed that it may increase androgen levels in the ovaries, resulting in a slowdown in the growth of (initial preantral) IP follicles, which may promote the development of more sinus follicles and make maturation more uniform, significantly increasing the number of follicles, recovered oocytes, MII oocytes, and fertilization rates in DOR patients (101). In terms of adjuvant therapy and drug combination, the current research is still limited, and we look forward to more breakthroughs in future research. Further details on the use and dosage of GH in the aforementioned studies are summarized in **Table 1**. However, the aforementioned clinical studies are mostly small-scale or single-center studies, which may be subject to bias and thus have certain limitations. Therefore, there is an urgent need to conduct larger-scale, multicenter randomized controlled trials (RCTs) to further validate the efficacy of GH therapy. Such trials are crucial for providing more robust and generalizable evidence for its clinical application.

**Table 1:** Use and Dosage of GH in Assisted Reproduction for DOR.

Trial	Study Design	Patient population	Intervention and Grouping	GH Dose and Duration	Outcomes following GH treatment.	Conclusion
Haydardedeoğlu 2015 (100)	Retrospective Cohort Study	DOR patients under the age of 40 with a baseline FSH level below 20 IU/L.	Experimental Group (DHEA, Transdermal Testosterone, and GH Combined Treatment Group): 33 patients, totaling 37 cycles. Control Group: 52 patients, totaling 102 conventional IVF/ICSI cycles.	A 12-week regimen of DHEA 25 mg/tid, combined with 25 mg transdermal testosterone for the last 4 weeks, and initiated 3 IU GH administration during the luteal phase.	The number of follicles, oocytes retrieved, MII oocytes, and fertilization rate were significantly increased; clinical pregnancy rate, ongoing pregnancy rate, and live birth rate were elevated; cycle cancellation rate decreased from 54.5% to 8.1%.	Even in patients with the poorest ovarian reserve, clinical pregnancy can be achieved by combining transdermal testosterone, DHEA, and GH.
Chen 2020 (98)	Retrospective Propensity Score-Matched Study	DOR patients who have not conceived in previous IVF cycles.	GH Group: 92 patients who received GH pretreatment. Control Group: 92 patients who did not receive GH pretreatment (matched to the GH group using propensity score matching (PSM) method on a 1:1 ratio based on age and body mass index (BMI)).	Recombinant Human Growth Hormone (rhGH) at a dosage of 2 IU per day for a duration of 4 weeks.	Following GH pretreatment, there was a significant increase in the number of retrieved oocytes and the number of transferable day-3 embryos; among women who conceived through embryo transfer (ET), the cumulative clinical pregnancy rate in the GH group was significantly higher than that in the control group.	In women with DOR, a 4-week course of GH pretreatment can enhance ovarian response to stimulation and improve IVF-ET outcomes.
Dogan 2021 (99)	Retrospective Cohort Study	Patients with DOR or POR, under the age of 40, who have not conceived in previous IVF cycles, have a baseline FSH of less than 20 IU/L, and possess complete clinical data.	GH Group: 54 patients receiving GH treatment. Control Group: 52 patients not receiving GH treatment.	During controlled ovarian stimulation (COS), a regimen of 4 IU GH per day for 8 consecutive days was administered.	On the day of HCG administration, the levels of E2 and P in the GH group were significantly higher than those in the control group. The number of retrieved oocytes and MII oocytes was slightly higher in the GH group compared to the control group, and the proportion of high-quality embryos was significantly higher in the GH group. After embryo transfer, the ongoing pregnancy rate and live birth rate in the GH group were significantly higher than those in the control group.	GH as an adjuvant therapy can improve ovarian response and IVF/ICSI outcomes, particularly in patients with DOR or POR.
He 2023 (96)	Prospective Observational Study	Patients with DOR are undergoing IVF cycles and employing minimal stimulation protocols for COS.	GH Group: 32patients receiving GH treatment. Control Group: 32 patients not receiving GH treatment.	During COS, a daily dose of 3 IU of rhGH was administered.	In the GH group, metabolic levels in follicular fluid were altered (glutathione ↑, shikimate ↑, itaconate ↑, SAM ↓), the number of retrieved oocytes significantly increased (correlated with itaconate ↑ and SAM ↓), and E2 levels and normal fertilization rates showed an upward trend.	GH may ameliorate ovarian response by reducing ROS through the elevation of shikimate and glutathione levels.
Zhang 2024 (97)	Retrospective Study	Patients with DOR aged 40 and below, who have a history of POR (retrieval of no more than three oocytes with conventional stimulation protocols), have normal chromosome karyotypes for both partners, possess complete clinical data, and undergo ART treatment.	GH1 Group (2-month pretreatment group): 53 patients GH2 Group (1-month pretreatment group): 400 patients	All DOR patients received GH 2 IU/day pretreatment upon entering the ovulation induction cycle, continuing until they met the conditions for ART. During the ovulation induction period, the daily dose of GH 2 IU/day was maintained until the day of HCG administration.	The number of retrieved oocytes in the GH pretreatment, GH1, GH2, and GH3 groups was significantly higher than in the control group (all P < 0.01). The number of retrieved oocytes in the GH1 and GH2 groups was similar and nominally higher than in the GH3 group. On the day of HCG administration, the E2 concentrations in the GH pretreatment, GH2, and GH3 groups were significantly higher than in the control group (all P < 0.01). In the GH1 group, 22 patients had more than one cycle of assisted reproductive treatment (without GH pretreatment) prior to receiving GH pretreatment; the number of retrieved oocytes in the GH pretreatment cycle was higher than in the non-GH pretreatment cycles, but the difference was not significant.	Extending the duration of GH pretreatment appropriately can increase the number of retrieved oocytes. In patients with DOR, GH pretreatment improved treatment outcomes. GH pretreatment exceeding one month did not increase the number of retrieved oocytes.

In the treatment of patients with DOR using GH, dosage and timing of administration are crucial. Multiple studies have shown that in ART, patients with poor ovarian response (POR) who receive 4 to 8 IU of GH daily during the follicular phase exhibit increased endometrial thickness, reduced gonadotropin requirements, and a higher number of mature oocytes compared to the control group (102). During the treatment period, patients should strictly follow medical advice for regular medication use and avoid arbitrary interruptions to ensure efficacy and safety. Dosage regimens need to be individually adjusted for different populations. For patients with DOR who have fertility needs, poor embryo quality, thin endometrium, and recurrent implantation failure, initiating GH treatment three months in advance can regulate follicle development from the preantral stage to maturity, thereby improving oocyte quality (103). For older patients with POR, a study involving women over 40 undergoing IVF demonstrated that appropriately increasing the GH dosage and extending the pretreatment period can improve pregnancy outcomes in patients with thin endometrium undergoing frozen embryo transfer (104). For patients with POR and recurrent implantation failure, the use of GH during follicle stimulation can mitigate the decline in ART efficiency due to aging, including improvements in the number of oocytes retrieved, fertilization rate, embryo quality, implantation rate, pregnancy rate, and live birth rate (6). In contrast, relatively younger patients (under 40 years old) with relatively better ovarian reserve can use a lower dose of 2 U/d and start pretreatment four weeks before ovulation induction (98). In summary, when treating patients with DOR using GH, the dosage and timing should be individually adjusted according to the specific circumstances of the patient to achieve the best therapeutic effect.

## 7 Potential adverse reactions and safety of GH therapy

Previous studies have indicated that long-term, high-dose use of GH in the treatment of DOR may trigger adverse reactions and potential risks across multiple systems. Specifically, GH can induce insulin resistance and glucose-lipid metabolic disorders by antagonizing insulin action, thereby increasing the risk of new-onset diabetes (105). Moreover, excessive use of GH may lead to cardiovascular complications such as acromegaly, myocardial hypertrophy, and hypertension through its effects on endothelial dysfunction, atherosclerosis formation, oxidative stress, and metabolic homeostasis (106).Although the direct association between GH and tumorigenesis has not been established, GH may increase the risk of tumor recurrence or proliferation (107). For example, one study found that over half of the tumor cells in 61% of cervical cancer patients express nuclear GHR, and high levels of nuclear GHR may act as a promoter of cervical cancer cell progression (108). However, another review summarizing the published papers to date concluded that GH use is safe in terms of tumor recurrence (109).Furthermore, excessive GH can also disrupt the stability of the reproductive endocrine axis. A study focusing on premenopausal women with acromegaly revealed that 64% of these women of childbearing age had low levels of AMH, suggesting that excessive GH may lead to a decrease in ovarian reserve (110).In summary, strict adherence to individualized principles is required in clinical applications, along with continuous monitoring of side effects and safety after medication use, to ensure the safety and efficacy of treatment.

## 8 Summary and outlook

The application of GH therapy in DOR has made significant progress, offering new therapeutic hope for many patients. DOR is a common reproductive disorder characterized by a reduction in the number and quality of ovarian follicles, leading to a decline in female fertility. As an important hormone for growth and metabolism regulation, GH promotes follicular development and improves oocyte quality through mechanisms such as the activation of the PI3K/Akt, Sirt3-Sod2, Notch-1, and JAK2-MAPK3/1 signaling pathways, thereby improving the fertility prognosis of DOR patients. Numerous clinical trials have confirmed the effectiveness of GH therapy for DOR in previous studies. GH can stimulate ovarian angiogenesis, improve the follicular microenvironment, and thereby promote follicular growth and development. Additionally, GH protects follicles from oxidative stress and other damages by regulating apoptotic processes, maintaining the health of the ovaries. However, issues regarding the optimal dosage, timing, and duration of GH therapy for DOR still require further exploration. Additionally, further investigation is required regarding the indications, contraindications, and potential adverse effects of GH therapy. Previous studies have indicated that long-term, high-dose use of GH may trigger adverse reactions and potential risks across multiple systems, including insulin resistance, cardiovascular complications, and potential impacts on tumor recurrence. Therefore, strict adherence to individualized principles is required in clinical applications, along with continuous monitoring of side effects and safety after medication use, to ensure the safety and efficacy of treatment. With the continuous advancement of technologies such as genomics and proteomics, we can gain a deeper understanding of the pathogenesis of DOR, thus providing more precise and personalized treatment plans for GH therapy. We anticipate that further basic and clinical research will validate and refine the methods and strategies for GH treatment of DOR.
